# Modern Sociology

**Published:** 1898-10-15

**Authors:** 


### Modern Sociology.

### PEOPLE'S BANKS.—III.

### Italian "Banche Popolari."

Among the disciples of Schultz was an Italian, Signor Luzzati, who has adapted the German principles to a Latin population. The fundamental idea of banking is more familiar to Italians than to most people; naturally enough, since it was to Italy that the science of banking owed its existence. The Banca Popolare of Milan, the original institution, has had many successors, all of which seem to bear a good reputation and do a large amount of work. It has been brought as an accusation against Luzzati, as against Schultz, that he does not provide for the banking requirements of the very poor, but when one notes that the majority of the loans granted by the "*banche popolari*" are for less than £40, many for only £10, and some for as little as eight shillings, the charge seems to fall to the ground. As a matter of fact, however, Luzzati does not, as a rule, grant long credit, so that he does not meet the requirements of poor agricultural borrowers, while he is of the greatest use to the small borrowers, mostly tradespeople, in towns. A great part of the work of these banks is the discounting of bills. A bill, as it is explained by Signor Ettore Levi, one of Luzzati's disciples, "stands for a productive transaction at its inception." A man, buying material for any manufacture, gives the seller of the raw material a bill, to be paid by him when he has made up and disposed of his goods, but for which the acceptor of the bill can obtain the money at once, subject only to a small discount. The convenience of this to both parties is obvious; but in this country it is hardly possible for a man in a small way of business to obtain such accommodation from any bank. Only large dealers can pay their accounts and obtain advances in that way. The merit of the People's Bank is that it gives the small man as good a chance as the big one. Of course, it is far more difficult to investigate the circumstances of a score of small traders than those of one large one; and, to meet this, we find in the Italian banks what is called a "*comitato di sconto*," a voluntary committee, who make a list of the various amounts which, in their opinion, the different members of the bank are "good for," and in accordance with that estimate discount their bills. It is open for a man to borrow on the joint security of himself and any of his friends who will back his bill, so long as the amount does not exceed the total amount for which the committee considers that the whole number have assets available.

Signor Luzzati, like Schultz, his master, issues shares, but, as a rule, the shares are small. On the other hand, they must be paid up promptly, on pain of forfeiture; and there is also an entrance fee. Thus in the original bank, that of Milan, the 50 lire (£2 share), and another £1 by way of entrance fee, must be paid up within 10 months. This may be too much for some who would gladly profit by banking facilities; but the difficulty thus created has been met by the establishment, around every large bank, of smaller ones, the shares in which are cheaper, in some cases standing as low as 5 lire (4s.) The strange thing is the confidence which these tiny institutions seem to inspire the public with. This is evidenced by the amount which they receive in deposits, from non-members as well as from their own shareholders. For example, in Montelupo, a poor district of Florence, the inhabitants of which are engaged in the making of lucifer matches, a bank was started; the shares were of 10 lire (8s.), to be paid up within 10 months. The shareholders numbered 375. Thus the original capital of the bank was £15, and when all the shares were paid up it amounted only to £150; yet in that first year of its existence it managed to attract deposits to the amount of £1,120, and lent out £1,240; and, as its profits from that business amounted to £120, the founders received greater part of their share capital back within the first year. Surely that is successful business!

It may be asked how so small a business, conducted by a few poor men, can win so much confidence. Signor Luzzati's answer is that it is done by "the capitalisation of honesty." This high-sounding phrase means that the greatest care is taken to lend only to respectable people, and to no larger amount than their assets, as calculated by the "*comitato di sconto*," will stand, and also to the fact that the affairs of the bank are always open to the public, a statement of them being published every month in a paper called the *Bolletino*. It is, of course, obvious that this high position has been won only by the exercise of the strictest probity, added to the best business management. What seems to us most remarkable is that this good management has been so generally available, even for concerns individually so small, though, in their totality, dealing with so very large sums. It must be remembered that even in the case of those banks which have now a large capital, and deal with larger loans, all have arisen from small beginnings. Even the parent bank, that of Milan, which has now a paid-up capital of £336,752, with a reserve of £168,376, when it first began operations in 1866 had for its total capital only £28, of which the largest single contribution was Signor Luzzati's own, the magnificent sum of 100 lire, or in our money £4. The success of the banks may be estimated from the fact that many of them pay 6, 8, 10, even 15 and 20 per cent. in dividend. Their founder has in vain protested against these large dividends; but he has at least succeeded in persuading them to give considerable sums to philanthropic objects, one of which, the "*prestito sull' onoro*," or "loan upon honour," has no parallel among us. These loans are granted to the very poor—but the respectable poor—who can offer no security. Very strange it seems, but it is true, that these loans are almost invariably repaid.

These *banche popolari* are founded, as we have said, on the Schultz model, and, like their originals, do not, it is admitted, meet the case of the small rural cultivators who are so numerous on the Continent. But, as in Germany, the want has been met by the "*casse rurali*" of Dr. Wollemborg, which are in almost every respect like the Raffeisen associations of which we have already spoken. These country banks have had the great advantage of the moral, and often the financial, support of the *banche popolari*, and in the comparatively short time—fifteen years—during which they have been established they have fought a good fight with the usurer, and made the peasant farmer in fact what he was often before only in name—the owner of his land and stock.

